# Accuracy of breast MRI in patients receiving neoadjuvant endocrine therapy: comprehensive imaging analysis and correlation with clinical and pathological assessments

**DOI:** 10.1007/s10549-020-05852-7

**Published:** 2020-08-12

**Authors:** Joana Reis, Jonas Christoffer Lindstrøm, Joao Boavida, Kjell-Inge Gjesdal, Daehoon Park, Nazli Bahrami, Manouchehr Seyedzadeh, Woldegabriel A. Melles, Torill Sauer, Jürgen Geisler, Jonn Terje Geitung

**Affiliations:** 1 Department of Diagnostic Imaging and Intervention, Akershus University Hospital (AHUS), Postboks 1000, 1478 Lørenskog, Norway; 2 Institute of Clinical Medicine, Campus AHUS, University of Oslo, Postboks 1000, 1478 Lørenskog, Norway; 3 Health Services Research Unit, Akershus University Hospital (AHUS), Postboks 1000, 1478 Lørenskog, Norway; 4 Sunnmøre MR-Clinic, Agrinorbygget, Langelansveg 15, 6010 Ålesund, Norway; 5 Department of Pathology, Oslo University Hospital (OUS), Postboks 4956, 0424 Nydalen, Norway; 6 Department of Breast and Endocrine Surgery, Akershus University Hospital (AHUS), Postboks 1000, 1478 Lørenskog, Norway; 7 Department of Oncology, Akershus University Hospital (AHUS), Postboks 1000, 1478 Lørenskog, Norway; 8 Translational Cancer Research Group, Akershus University Hospital (AHUS), Postboks 1000, 1478 Lørenskog, Norway; 9 Department of Pathology, Akershus University Hospital (AHUS), Postboks 1000, 1478 Lørenskog, Norway

**Keywords:** Breast cancer, Neoadjuvant therapy, Magnetic resonance, Tumour response

## Abstract

**Purpose:**

To assess the accuracy of magnetic resonance imaging (MRI) measurements in locally advanced oestrogen receptor-positive and human epidermal growth factor receptor 2-negative breast tumours before, during and after neoadjuvant endocrine treatment (NET) for evaluation of tumour response in comparison with clinical and pathological assessments.

**Methods:**

This prospective study enrolled postmenopausal patients treated neoadjuvant with letrozole and exemestane given sequentially in an intra-patient cross-over regimen. Fifty-four patients were initially recruited, but only 35 fulfilled the inclusion criteria and confirmed to participate with a median age of 77. Tumours were scanned with MRI prior to treatment, during the eighth week of treatment and prior to surgery. Additionally, changes in longest diameter on clinical examination (CE) and tumour size at pathology were determined. Pre- and post-operative measurements of tumour size were compared in order to evaluate tumour response.

**Results:**

The correlation between post-treatment MRI size and pathology was moderate and higher with a correlation coefficient (*r*) 0.64 compared to the correlation between CE and pathology *r* = 0.25. Post-treatment MRI and clinical results had a negligible bias towards underestimation of lesion size. Tumour size on MRI and CE had 0.82 cm and 0.52 cm lower mean size than tumour size measured by pathology, respectively.

**Conclusions:**

The higher correlation between measurements of residual disease obtained on MRI and those obtained with pathology validates the accuracy of imaging assessment during NET. MRI was found to be more accurate for estimating complete responses than clinical assessments and warrants further investigation in larger cohorts to validate this finding.

**Electronic supplementary material:**

The online version of this article (10.1007/s10549-020-05852-7) contains supplementary material, which is available to authorized users.

## Introduction

Current response assessments of locally advanced breast cancer (LABC) in the neoadjuvant setting are not entirely accurate in determining pathological complete response, depending on the modality used, the measurement technique and varied response of different tumour subtypes [1, 2]. Tailoring the most accurate tumour size after neoadjuvant treatment must require a precise assessment, in order to achieve the most appropriate surgical management: mastectomy or breast-conserving surgery (BCS). Conventional assessment includes clinical examination (CE) and imaging modalities (mammography, ultrasound and/or magnetic resonance imaging (MRI)), along with pathological examinations of the sectioned surgical specimen, which take place typically during the first weeks after surgery.

Performed by a trained physician, CE is a widely recognized assessment for detecting breast cancers and for tumour size monitoring [3]. CE is a non-invasive, low-cost method, without the use of ionizing radiation [2, 4]. For patients with breast symptoms, a CE should be performed before any additional imaging assessments are sought. Additionally, it should be a part of routine periodic examinations, especially in older women (more than 69 years old) and in women less than 50 years who are less likely to undergo mammographic screening [3]. CE is typically performed prior to each neoadjuvant therapy cycle or every 4–6 weeks during neoadjuvant endocrine therapy (NET).

Although mammography and ultrasound are reliable methods to analyse tumour size at diagnosis, changes within the tumour after neoadjuvant therapy may be difficult to assess. Targeted ultrasound is mandatory for malignant breast tumours and of the axilla for clinical staging if neoadjuvant therapy is planned [2]. Ultrasound has been revealed to be a better predictor for pathologic tumour size than mammography after treatment with neoadjuvant therapy. However, ultrasound is operator dependent, and its accuracy varies [2, 5, 6]. Mammography has been less specific than CE for detecting presence of residual tumour after therapy, and it adds challenges with mammographically occult tumours and microcalcifications which do not correlate with presence of viable tumours [2]. Furthermore, mammography has the disadvantage of being less accurate in high density breasts.

Dynamic contrast-enhanced MRI is the most sensitive of available imaging modalities in characterization of breast malignant tumours as a functional technique that allows the evaluation of residual viable tumour after neoadjuvant therapy by detecting changes in tumour vascularity [7–14]. Multiple studies have clarified that breast MRI is the most accurate imaging modality to determine disease response to neoadjuvant chemotherapy, with sensitivity approaching 90% and specificity of 60% to 100%, and it is particularly helpful in patients with confirmed multifocal and multicentric tumours on the pre-treatment study [1, 2, 4, 7, 8, 15–17]. Breast MRI has been considered to have a role in guiding breast cancer surgery by differentiating residual tumour from pathological complete response after neoadjuvant therapy, and planning resections to achieve clear margins in BCS [11, 18].

Response Evaluation Criteria in Solid Tumours (RECIST) guidelines recommend assessment of the largest tumour diameter [19]. Overestimation of tumour size may lead to exceedingly radical surgery and poorer cosmetic and psychosocial outcomes; underestimation may lead to involved surgical margins, and apparent radical surgery, though with missed additional tumour foci, eventually leading to a reoperation [20].

No previous research has investigated the performance of clinical and MRI assessment methods in the evaluation of residual tumour size in patients receiving combined neoadjuvant endocrine therapy with third generation aromatase inhibitors [21, 22]. Aromatase inhibitors (AIs) are well-established and low-toxicity drugs to improve surgical outcomes in LABC in elderly or otherwise fragile and postmenopausal patients, particularly in selected patients with oestrogen receptor (ER) highly (> 50%) positive disease [23–28]. Compared to neoadjuvant chemotherapy, AIs have demonstrated comparable outcomes in postmenopausal patients with ER positive LABC [29–32]. Likewise, several clinical randomized trials have confirmed that third generations AIs (letrozole, anastrozole, exemestane) are preferred over ER antagonists such as tamoxifen, due to higher clinical and radiological response rates [33–35]. Nonetheless, tumours respond differently to neoadjuvant AIs and not all patients respond in a satisfactory way, sometimes even warranting additional neoadjuvant chemotherapy.

The current lack of published studies to assess the accuracy of MRI in predicting residual size of breast tumours following NET opens opportunities for evidence-based studies [10]. There have been many published studies determining the diagnostic advantages of MRI versus clinical current assessments to evaluate the tumour response after neoadjuvant chemotherapy. Although, it is important to understand the different mechanisms of action between chemotherapy and endocrine therapy, and to recognize that tumours profiles and tumours stages are distinct. As neoadjuvant therapy, especially endocrine therapy, for breast cancer continues to move beyond locally advanced disease, efforts such as this one will be imperative to guide daily clinical practice, embracing both the available high-level evidence and guidelines to investigate current assessments for evaluating tumour response to neoadjuvant therapy.

The aim of the current study is to compare tumour size estimated by CE and MRI with the tumour size in surgical specimen, in order to determine which is the most accurate assessment to evaluate tumour response in patients with LABC treated neoadjuvant with aromatase inhibitor (NAAI) therapy.

## Materials and methods

### study population

**Fig. 1:**
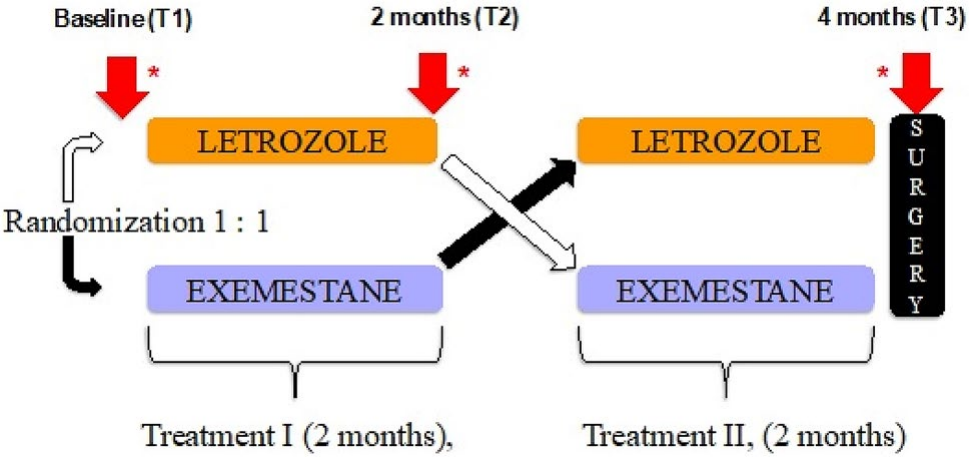
Design of the Neoletexe trial: a prospective, randomized intra-patient cross-over study. *Time points for breast magnetic resonance imaging examinations, open surgical biopsies of breast tumour (acquired after MRI examinations at baseline and after 2 months of treatment; and the third biopsy after 4 months of treatment and after surgery) and blood samples

**Fig. 2:**
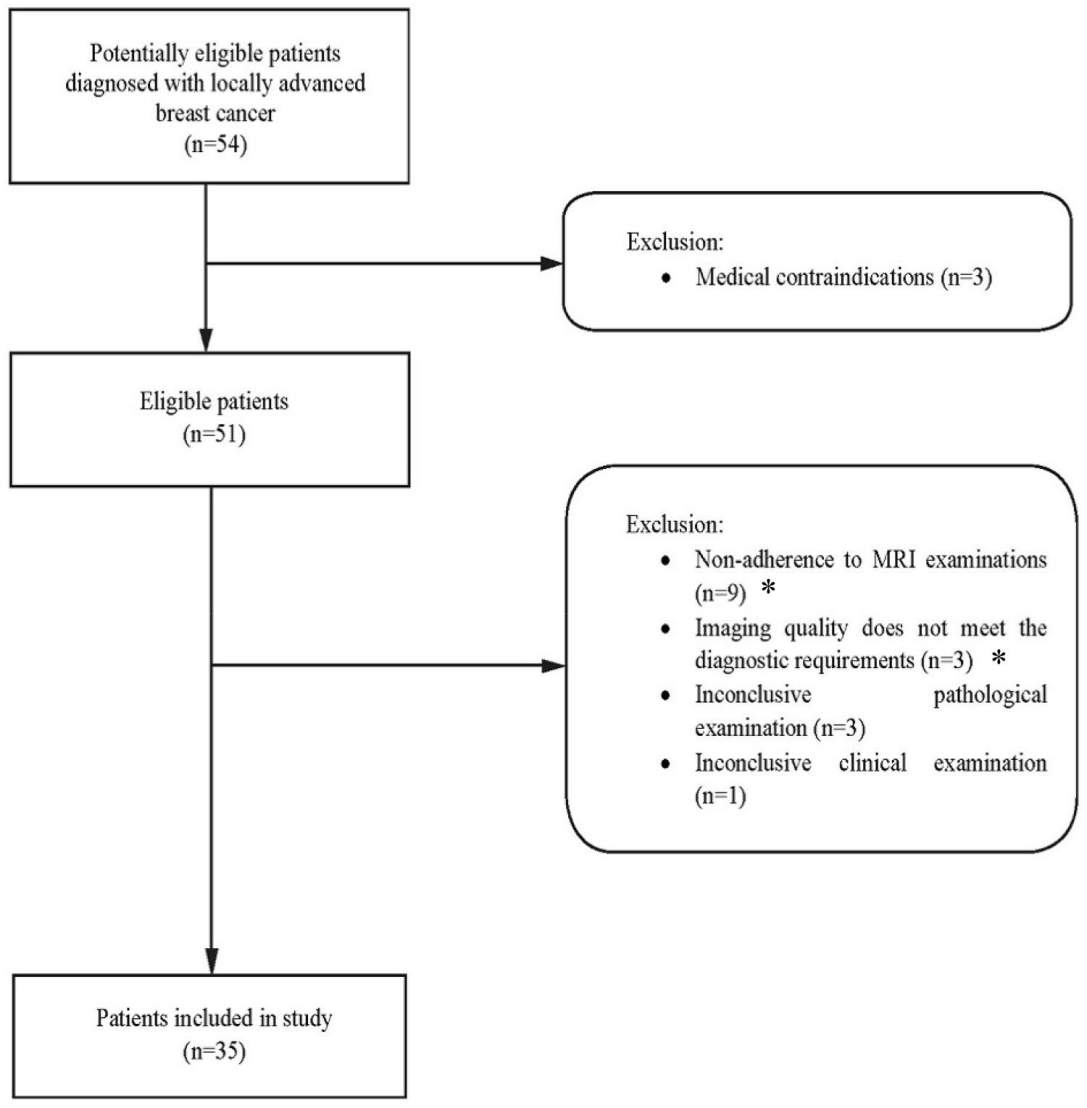
Flowchart of participants through the study including the patient’s enrolment and exclusion criteria. *Non-adherence to MRI examinations: refusal MRI or refusal to complete MRI examination (e.g. claustrophobia, and/or anxiety); imaging quality does not meet the diagnostic requirements: poor image quality (e.g. motion artefacts, chemical shift artefacts, and inadequate image contrast). *LD* Longest diameter, *MRI* magnetic resonance imaging, *n* number of patients

From February 2015 to November 2019, patients with biopsy-proven locally advanced oestrogen receptor (ER)-positive breast cancer and human epidermal growth factor receptor 2 (HER-2)-negative status suitable for NET were eligible for this prospective, open-label, randomized sub-study of the Neoletexe trial, (Fig. 1) [36]. All participants had to be postmenopausal to benefit from aromatase inhibition. Figure 2 reports a flowchart of the study cohort in which 54 patients were initially recruited, but only 35 fulfilled the inclusion criteria and confirmed to participate. Thirty-four of the patients studied were female and only one male with a total median age at diagnosis of 77 years, and a mean age of 74.3 years.

The exclusion criteria were triple-negative breast cancer, life-threatening metastasis, previous therapy for breast cancer within the last 12 months and/or medications that may interfere with endocrine therapy, inconclusive imaging, oncological and/or pathological measurements (Supplementary Table 1).

The diagnosis of breast cancer was established after a digital mammography, accompanied by breast and axillary ultrasound, a stereotactic or ultrasound- guided breast biopsy and CE. Primary tumours were classified using clinical and radiological tumour, node and metastasis (TNM) staging system for breast cancer, according to the classification system proposed by the American Joint Committee on Cancer (AJCC, 2017) and Union for International Cancer Control (UICC, 2017). All patients, as part of their clinical care, were screened for distant metastasis with thoracic, abdominal and pelvic computed tomography scan and bone scintigraphy. Only two patients presented with limited systemic metastasis at baseline were allowed to enrol. Table 1 includes the characteristics of included patients (*n* = 35).

**Table 1:** Characteristics of the analysis group (*n* = 35)

Characteristics	Analysis group (*n* = 35)	
	Baseline	Postoperative
Age		
Median	77	
Gender		
Female	34	
Male	1	
Menopausal/andropause status		
Postmenopausal	34	
Andropause	1	
Clinical exam		NA
Palpable	35	
Skin retraction		NA
Yes	20	
No	15	
Nipple retraction		NA
Yes	12	
No	23	
Skin thickening		NA
Yes	23	
No	12	
Clinical tumour stage		NA
T2	0	
T3	6	
T4	29	
Clinical nodal status		NA
N0	28	
N1	7	
Imaging (MRI) tumour stage		NA
T2	1	
T3	4	
T4	30	
Imaging (MRI) nodal status		NA
N0	25	
N1	9	
N2	1	
Oestrogen receptor status		
Positive	35	3
Negative	0	0
Analysis not performed	0	2
Progesterone receptor status		
Positive	29	28
Negative	6	5
Analysis not performed	0	2
HER-2 status		
Negative	35	33
Positive	0	0
Analysis not performed	0	2
Invasive histologic findings		
Ductal carcinoma, NST	28	23
Lobular carcinoma	5	8
Papillary	1	0
Mucinous	1	1
pCR	NA	3
DCIS present (*)		
No	32	21
Yes	3	14
LCIS present (*)		
No	34	30
Yes	1	5
Surgery type	NA	
Breast- conserving surgery		2
Mastectomy		33
Pathologic tumour stage	NA	
T0		2
T1		8
T2		22
T3		2
Tis		1
Pathologic nodal status	NA	
N0		15
N1		15
N2		5

*CT* Computed tomography, *DCIS* ductal carcinoma in situ, *HER-2* human epidermal growth factor receptor 2, *n* number of patients, *NA* non-applicable, *NST* non-specific type, *LCIS* lobular carcinoma in situ, *pCR* pathologic complete response

^*^In situ component at baseline was obtained based on diagnostic biopsy samples and at the post-operative time point based on full specimens

### Imaging

MRI scans were performed prior to the start of NAAI therapy (baseline), 8 weeks after the first cycle and prior to the second cycle of therapy (second examination), after the final administration of NAAI therapy and immediately before surgery (third examination) (Fig. 1). Imaging was acquired using a Philips Ingenia 1.5 T scan (Philips Healthcare, Best, Netherlands). A dedicated sixteen- channel bilateral breast coil with parallel imaging capabilities was applied. The MRI protocol consisted prior to the administration of contrast of an axial turbo spin echo (TSE) T1-weighted sequence, an axial single-shot echo planar (SS-EPI) diffusion-weighted imaging with two respective b factors (50, 800) (DWI SSh-EPI) and a axial 3-dimensional (3D) T2-weighted with fat suppression. Two dynamic sequences where then applied in an interleaved pattern prior (for baseline acquisition data) and during the injection of the contrast agent. The high temporal resolution images were acquired using a 3D TI_T2* weighted multi-echo planar imaging (EPI) sequence and intercalated with a dynamic high spatial resolution 3D T1-weighted turbo field echo (TFE) sequence. Both contrast- enhanced imaging sequences include a total scanning time of 7 min with a full coverage of both breasts with no slice gap. More details on the breast MRI sequences are provided in Table 2. Diagnostic mammography and ultrasound examinations were performed at baseline according to the routine practice of our institution; mammographic and sonographic results were therefore not included in the comparison analysis. The institutions breast imaging radiologists interpreted these examinations, according to the American College of Radiology Breast Imaging- Reporting and Data System (BI-RADS®) lexicon (ACR BI-RADS® Atlas 2013, https://www.acr.org/Clinical-Resources/Reporting-and-Data-Systems/Bi-Rads).

**Table 2:** Technical parameters utilized for breast magnetic resonance sequences acquisition (Philips)

Sequence	TR/TE (ms)	Flip angle	Dyn Reps	NSA	FOV (mm)	Slice thickness (mm)	Acquisition time
Pre- contrast							
T1W-TSE	487/8	NA	1	2	280 × 340	3	2.40 min
DWI SSh SE- EPI	7000/103	NA	1	3	280 × 340	3	5.50 min
FS 3DT2W	1300/145	NA	1	1	370 × 370	2.2	5.20 min
After a single injection of CA							
3DT1_T2* multi-echo EPI	38/6.2/8.8	28º	41	1	280 × 340	8	2,84 s/volume
3D T1W THRIVE	5.4/2.6	12 º	6	1	360 × 120	2	56,5 s/volume

*CA* Contrast agent, *DWI SSh SE- EPI* diffusion-weighted imaging, single- shot, echo planar imaging with two respective b factors (50, 800), *T1W THRIVE* high spatial resolution 3D T1-weighted turbo field echo (TFE) sequence, *EPI* echo planar imaging, *FOV* field of view, *NA* non-applicable, *NSA* number of signals, *FS* fat suppression, *TE* time echo, *TR* repetition time, *TSE* turbo spin echo, *T1W* T1- weighted, *T2W* T2- weighted, *3D* 3-dimensional

### Clinical assessment

Clinical tumour assessments were conducted at the oncological outpatient clinic by the same medical breast cancer oncologist every 4 weeks during the entire study, but only evaluations performed at baseline, following 8 weeks and 16 weeks were included in this analysis. The NAAI intra-patient cross-over regimen consisted of one of the following treatment arms: letrozole 2.5 mg o.d. for at least 8 weeks thereafter continuing with exemestane 25 mg o.d. for another 8 weeks prior to surgery; and exemestane 25 mg o.d. for at least 8 weeks thereafter continuing with letrozole 2.5 mg o.d. for 8 weeks prior to surgery (Fig. 1) [36]. Calliper measurements of maximum tumour size (in centimetres) and tumour quadrant location were recorded. Afterwards, response categories were assessed and categorized into four groups: complete response, partial response, progressive and stable disease. Complete clinical response was defined as no palpable lesions. Partial clinical response was defined as a decrease in the longest diameter (LD) of primary tumour of at least a 30%. Progressive disease was defined as at least a 20% increase in the LD of the primary tumour. Stable disease was defined as neither partial response nor progressive disease. Collection of blood samples was performed before each time point (Fig. 1). Upon completion of NAAI therapy, all patients underwent surgery following CE by an experienced breast surgeon, and treated with adjuvant therapy using letrozole 2.5 mg o.d. for a period of at least 5 years as standard recommendation. Patients with progressive or stable response during or after NAAI therapy were offered additional treatment options according to the national guidelines without any restriction.

### Image analysis

**Fig. 3:**
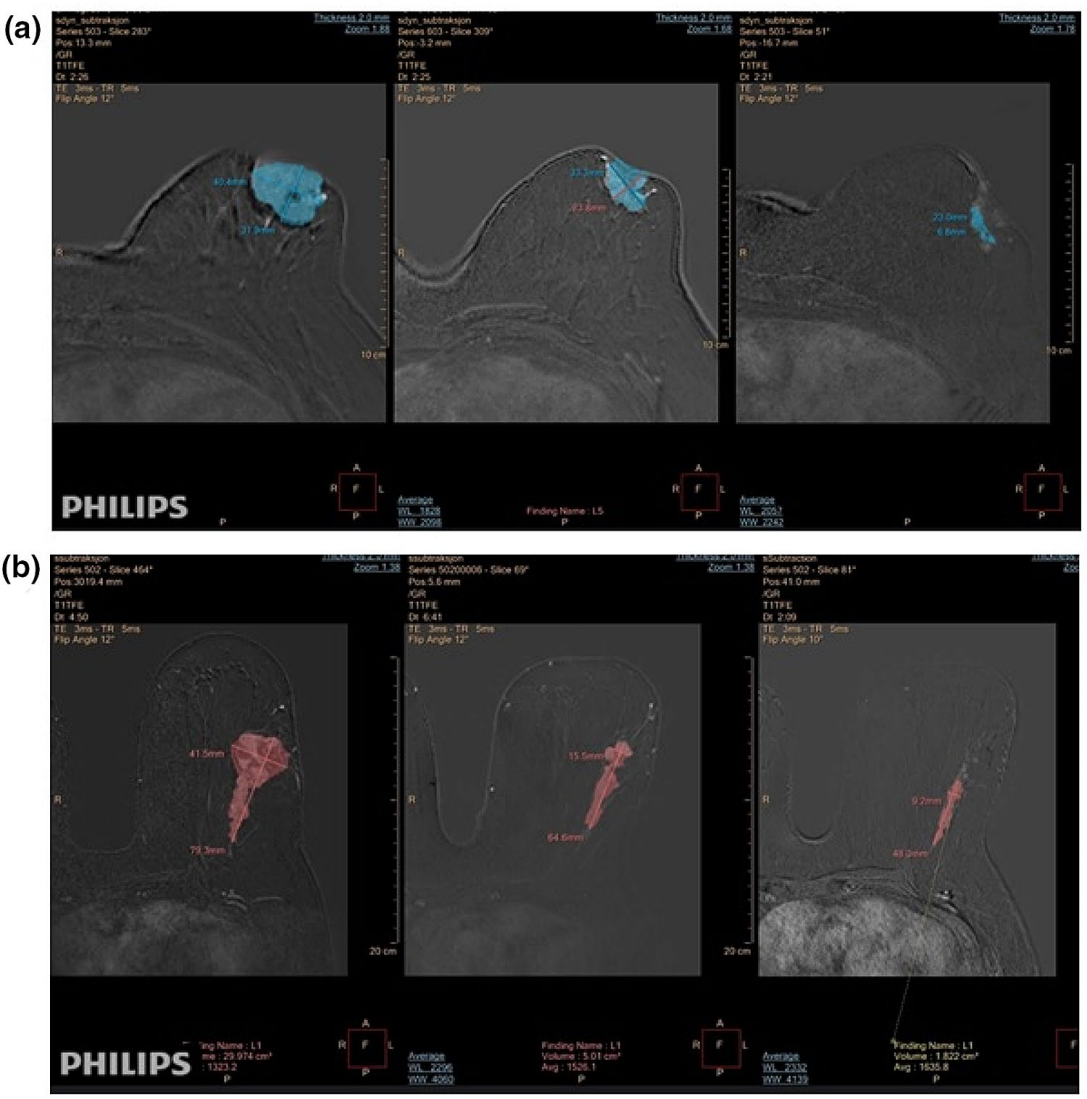
**a**, **b** Two different patients’ magnetic resonance imaging examinations demonstrating response to treatment and residual tumour sizes. Manual assessment of tumour longest diameter using the tumour tracking system incorporated in Philips IntelliSpace Portal program. Description of the time points from left to right: at baseline, between regimens (after 2 months of treatment) and presurgery (after 4 months of treatment)

Image evaluation included both qualitative, consistent with the standard BI-RADS® and TNM classifications, and quantitative imaging interpretations. All images were independently analysed by two breast imaging radiologists (one with more than 10 years and the other with 2 years of experience in interpreting breast MRI), and reviewed afterwards in a consensus interpretation. The radiologists were blinded to findings from clinical assessment and were not aware of the post-operative histopathology results. Table 3 shows the qualitative MRI findings at baseline. LD was measured as the greatest extent of disease. Response categories based on RECIST were assessed and categorized into four groups: complete response (no enhancement of all malignant lesions), partial response (decrease in the LD of primary tumour of at least a 30%), progressive disease (at least a 20% increase in the LD of the primary tumour) and stable disease (no change). Contrast- enhanced images were analysed using Philips IntelliSpace Portal program (Philips Healthcare, The Netherlands). Manual assessment of tumour LD was performed through the tumour tracking software incorporated in Philips IntelliSpace Portal program at the point at maximum tumour enhancement (Fig. 3).

**Table 3:** Few MRI findings based on ACR BI-RADS® Atlas Fifth Edition at baseline. Diverse associated features could be present at the same MRI

MRI findings	Analysis group (*n* = 35)
Mass	27
Shape	
Oval	1
Round	0
Irregular	26
Margin	
Circumscribed	1
Not circumscribed	26
Internal enhancement characteristics	
Homogeneous	0
Heterogeneous	27
Rim enhancement	0
Dark internal septations	0
Non- mass enhancement	4
Non- mass enhancement and mass	4
Associated features	
Nipple retraction	14
Nipple invasion	3
Skin retraction	13
Skin thickening	15
Skin invasion	10
Axillary adenopathy	10
Pectoralis muscle invasion	5
Chest wall invasion	0
Architectural distortion	0

*ACR* American college radiology, *BI-RADS®* breast imaging-reporting and data system, *MRI* magnetic resonance imaging, *n* number of patients

### Pathological assessment

Pathology was used as the gold standard for comparing tumour size measurements after neoadjuvant treatment. Comprehensive pathological analysis of surgical specimens was performed by an experienced breast cancer pathologist. During the course of the study supplementary scientific incisional surgical biopsies of tumour tissue were obtained, after the MRI examinations, at baseline, after 8 weeks and after surgery (Fig. 1). These scientific biopsies allowed verification of ER status, progesterone receptor (PR) status and HER-2 status.

Pathological response was defined as complete responder (no residual invasive disease was present) or not complete responder (residual invasive disease present). Total extent of residual disease was reported, measured as the greatest one- dimensional extent in centimetres of residual invasive cancer including intervening areas of fibrosis and/or necrosis. In situ components measurements were not included.

### Statistical analysis

Descriptive statistics were used to compared two measurements of tumour response (changes in LD at MRI and CE) to predict pathologic outcome. Each predictor variable was measured at three time points. Missing data elements were not included in the analysis. The Spearman rank correlation was applied to study the statistical dependence between the classification of the tumour sizes estimated by CE and MRI, and pathological results. The Bland–Altman analysis was used to evaluate deviations from the mean of the measures by two methods (CE versus pathology, and MRI versus pathology) and to estimate a range of agreement, within which 95% of the differences between the measurement by CE or MRI and the pathological measurement. Box plots were conducted to visually summarized the distribution of the numerical data. Statistical analysis was performed using R software. A two-sided *p* ≤ 0.05 was considered statistically significant.

### Ethical requirements

The Regional Committee for Medical and Health Research Ethics South-East Norway approved the study protocol. Informed consent was obtained from all individual participants included in the study. The imaging findings have not previously been reported.

## Results

The clinical-based TNM classification was used to identify 29 cases of T4 tumours (82.9%) and 6 cases of T3 tumours (17.1%) (Table 1).

### Baseline comparison of tumour size before starting therapy

**Fig. 4:**
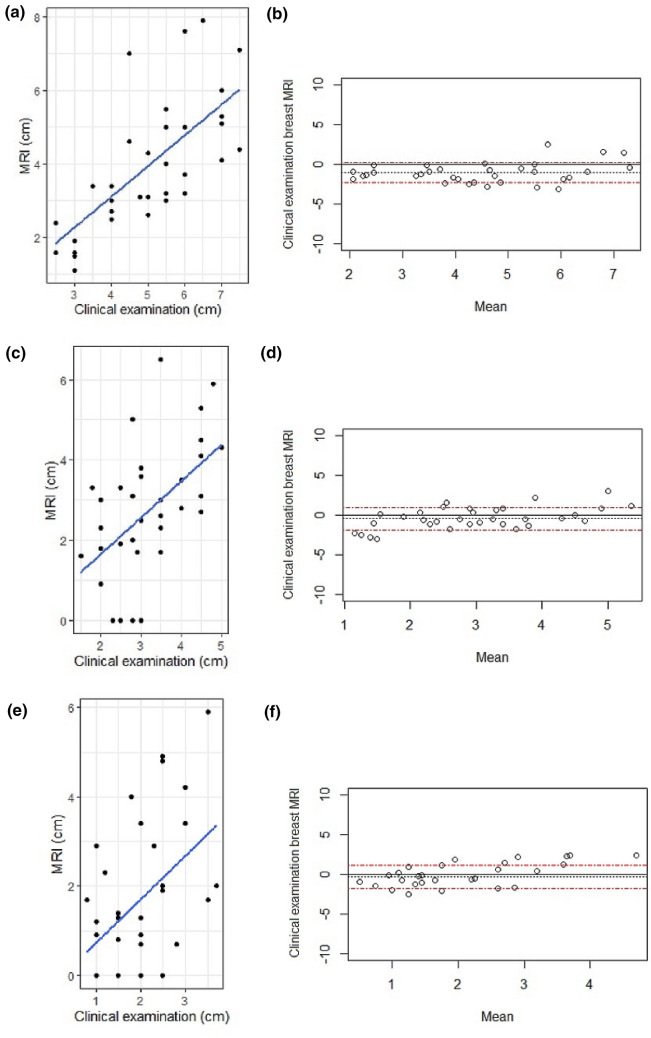
Scatterplots showing the longest diameter of tumour measured on MRI and CE, and Bland–Altman plots illustrating the size difference between the measurements by MRI and CE (**a**–**f**). **a** Scatterplot at baseline, **b** Bland–Altman plot at baseline, **c** Scatterplot between regimens, **d** Bland–Altman plot between regimens, **e** Scatterplot at the end of the neoadjuvant therapy and **f** Bland–Altman plot at the end of the neoadjuvant therapy. Correlation between measurements at baseline was strong *r* = 0.71, between measurements was moderate *r* = 0.53 and at the end of the neoadjuvant therapy was moderate r = 0.46. Trend line (*black line*) depicts least-squares fit for data. MRI: magnetic resonance imaging

The median tumour size (LD) at baseline on MRI was 3.4 cm (interquartile range 2.9–5.0 cm); on CE was 5.0 cm (interquartile range 4.0–6.0 cm). Spearman correlation showed that baseline tumour size on CE and MRI had a strong positive agreement (*r* = 0.71), (Fig. 4a). The Bland–Altman plot was used to measure the difference of pre-surgical tumour sizes against the mean value of MRI and clinical assessments. The Bland–Altman (Fig. 4b) analysis indicated that the average lesion size estimated by MRI was smaller than compared with CE. The mean difference between the two measurements was 1.06 cm; with a 95% confidence interval (CI) of 0.63–1.49 cm (*p* ≤ 0).

### Between- regimens time point comparison of tumour size

The median tumour size between regimens on MRI was 2.7 cm (interquartile range 1.7–3.6 cm); on CE was 3.0 cm (interquartile range 2.5–3.8 cm). Spearman correlation showed that at this time point tumour size on CE and MRI had a moderate positive agreement (*r* = 0.53), (Fig. 4c). The Bland–Altman (Fig. 4d) analysis suggested that average size estimated by MRI was greater in larger tumours and lesser in smaller tumours compared with CE (mean difference 0.45 cm, CI of − 0.04 to 0.94 cm, *p* = 0.07).

### Comparison of tumour size after completion of therapy

The median size of the tumours after completion of NAAI therapy on MRI was 1.3 cm (interquartile range 0.4–2.6 cm) and on CE 2.0 cm (interquartile range 1.5–2.5 cm), with a moderate agreement, *r* = 0.46, (Fig. 4e). The Bland–Altman (Fig. 4f) analysis suggested that the average lesion size estimated by MRI was larger than compared with CE (mean difference 0.30 cm, with a 95% CI of − 0.20 to 0.80 cm, *p* = 0.23).

### Assessments outcomes and response evaluation

**Fig. 5:**
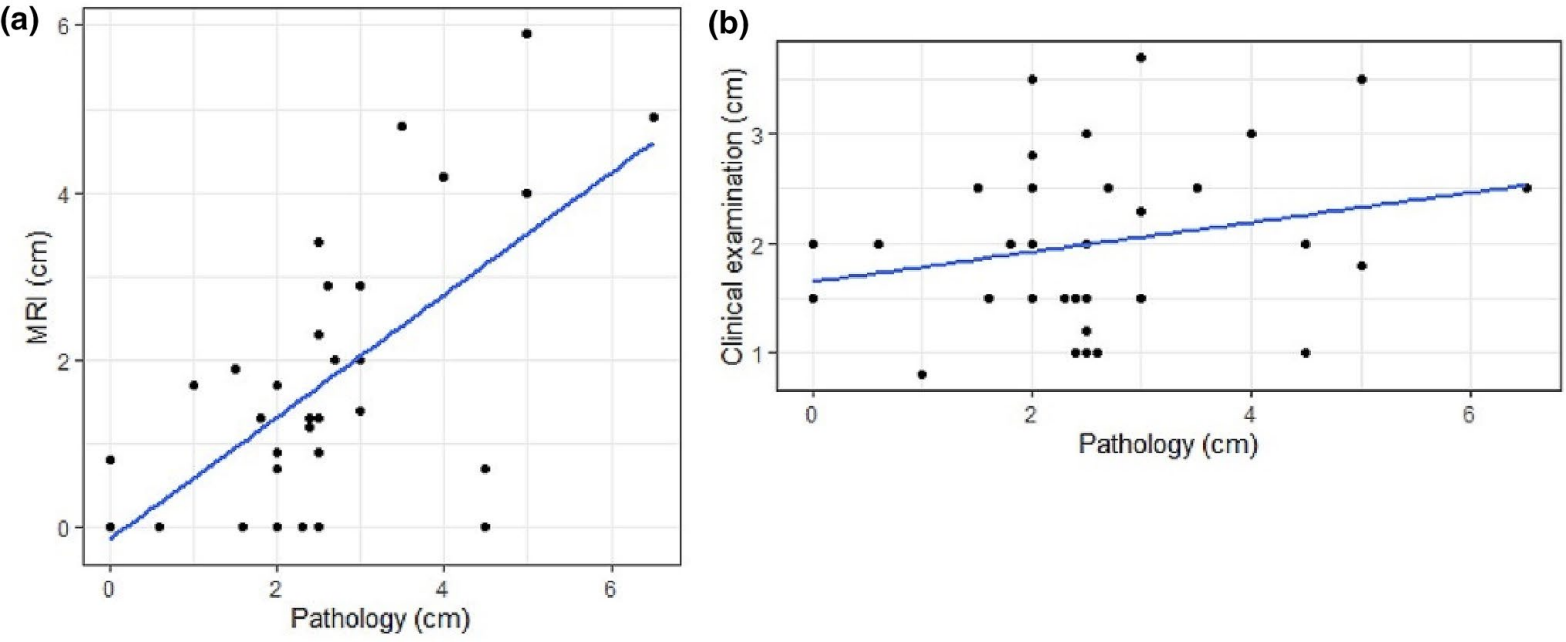
**a**, **b** Scatterplots showing longest diameter of tumour measured on MRI and pathology **a** and by CE and pathology **b** at the the end of the neoadjuvant therapy and after surgery. Correlation between MRI and pathology was moderate and stronger *r* = 0.64, then between CE and pathology, *r* = 0.25. *MRI* Magnetic resonance imaging

Pathological tumour size median was 2.5 cm (interquartile range 2.0–3.0 cm). The correlation between post-treatment MRI size and pathology was moderate and higher (*r* = 0.64, *p* = 0.001) compared to the correlation between CE and pathology (*r* = 0.25, *p* = 0.04), (Fig. 5). Tumour size on MRI had 0.82 cm lower mean size (95% CI of 0.37–1.27 cm) than tumour size measured by pathology. For CE, the measured tumour size was on average 0.52 cm smaller (95% CI of 0.03–1.02 cm).

**Fig. 6:**
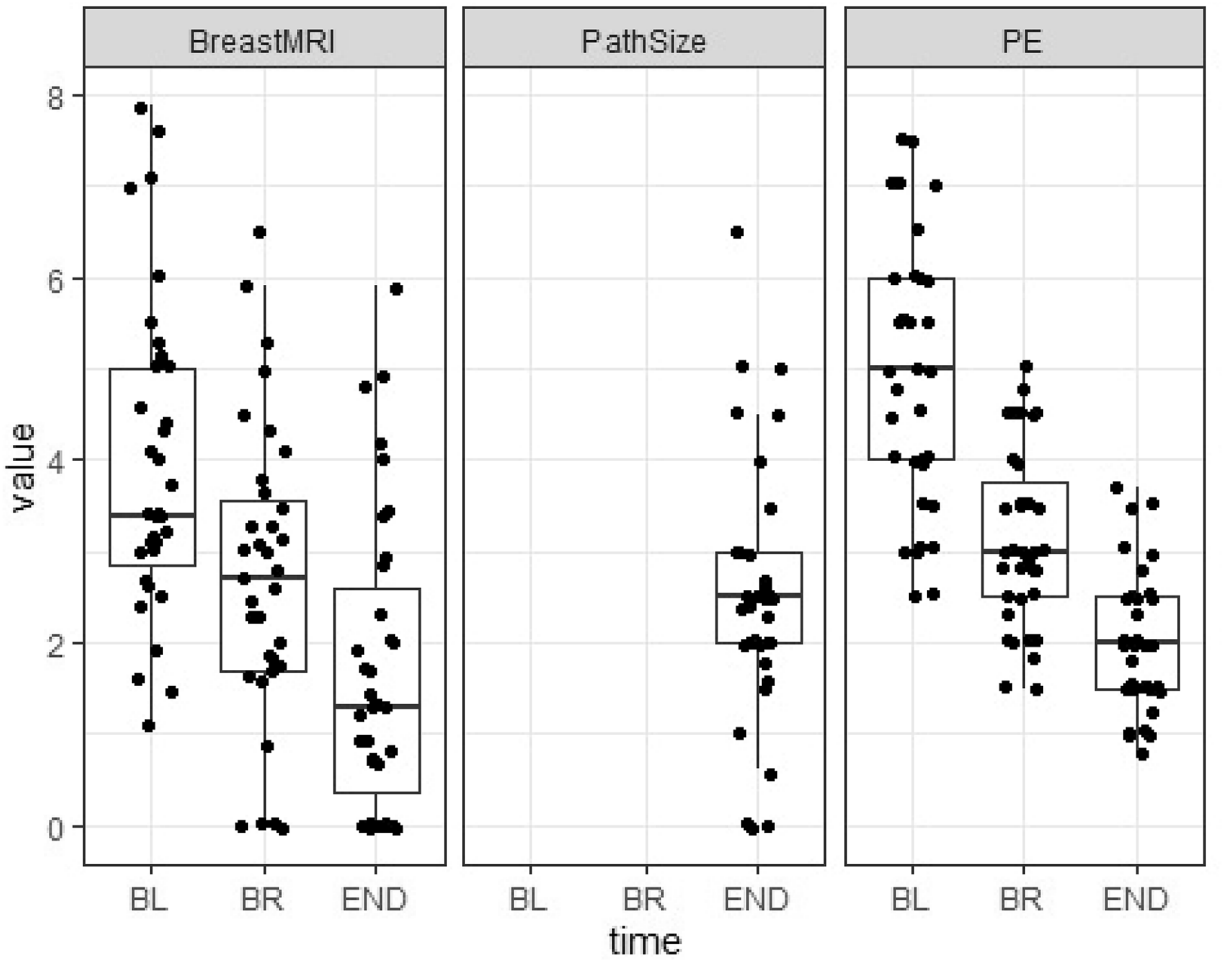
Comparison of the residual tumour size at baseline, between regimens and at the end of neoadjuvant therapy on MRI and CE. In addition, comparison of residual tumour size at the end of neoadjuvant therapy between MRI, pathological and clinical measurements. Box plots illustrating the median size of the three variables, with the corresponding interquartile range and whiskers from the 5th to the 95th percentile. *BL* Baseline, *BR* between regimens, *END* the end of therapy, *MRI* magnetic resonance imaging, *PathSize* pathological size, *PE* physical/CE

Figure 6 shows boxplots of all three measurement variables. MRI underestimated tumour size in 24 patients (68.6%) and overestimated in 9 patients (25.7%) compared to pathology. At completion of neoadjuvant therapy, 35 (100%) patients in the analysis showed a clinical partial response, and in turn MRI demonstrated a partial response in 25 (71.4%) patients, complete response in 9 (25.7%) patients and stable disease in 1 (2.9%). Thirty-two (91.4%) patients in the pathological analysis were not complete responders and just 3 (8.6%) patients were reported as complete pathological responders. Two of the three complete responses that were determined on pathology showed complete response at MRI. In addition, all of the three pathological complete responders were found to have clinical residual disease.

## Discussion

It is of vital importance to develop effective assessments of treatment response to maximize patient benefit during neoadjuvant breast cancer therapy. The results of this study indicate that breast MRI is an accurate method for assessing early response evaluation, residual disease and surgical planning in the neoadjuvant setting. In comparison with pathology and CE, the diagnostic accuracy of MRI is stronger in assessing disease extent, screening for other foci either in the affected or in the contralateral breast, and investigating satellite tumours as a repeatable 3D assessment [37, 38]. Regardless significant improvement in MRI technique, interpretation and analysis, tumour size can be over- or underestimated [17, 39–41]. Although, unlike pathological response, early changes in tumour measures by MRI can be assessed at a stage when treatment regimen can still be modified. Pathological response is strictly an endpoint and cannot be used to improve treatment planning [16, 42].

Imaging measurements are aggregations of all tumour tissue that meet criteria for signal enhancement and are dependent on parameters of the image’s acquisitions. These parameters include timing relative to contrast agent injection and physiologic conditions affecting the distribution of the contrast agent.

Furthermore, the effects in tumour vascularity induced by anti- angiogenetic effects in response to neoadjuvant therapy may explain the decrease or delay in imaging enhancement, demonstrating greater bias in residual tumour measurement. Reactive inflammation, fibrosis or necrosis are difficult to distinguish from residual tumour, and measurement errors may be cumulative when tumours regress as multiple, scattered deposits [19, 43].

In our sample population MRI tended to underestimate the actual tumour size in 68.6% of the cases and overestimated the pathology size in 25.7% of the cases directly prior to surgery.

Seven cases were discordantly diagnosed as imaging complete response but non-pathological complete response after NAAI therapy. The decrease in enhancement secondary to the changes in tumour vascularization induced by the anti-angiogenetic effect of therapeutic agents also contributed to the underestimation of MRI in the prediction of residual tumour size, and a high rate of false- negative interpretations [44]. After therapy, an enhanced area was no longer detected by MRI thus, the tumour response was considered complete. However, pathological analysis of the surgical specimen revealed microscopically scattered residual cancer nests in the tumour bed, indicating a non-pathological complete response.

The overestimation percentage is consistent with the literature, ranging from 6 to 81% [45–47]. We found that MRI diagnosed only one case of pathological complete response as a non-imaging complete response. Fibrous granulation tissue that may have caused the misdiagnosis that was observed in that case. MRI after NAAI therapy showed a small mass measuring less than 1 cm in diameter with scattered enhancement, interpreted as an imaging non-complete response. Pathology, however, showed fibrous granulation tissue in the surgical specimen without residual cancer cells, indicating pathological complete response. The fibrous granulation tissue contained inflammatory cells and numerous small vessels.

Mennella et al. found that the main reason for discordance between MRI and pathological measures is ductal carcinoma in situ (DCIS) [48]. MRI measurement included both invasive and non-invasive components, while it is well accepted that pathologists consider just the invasive component size of the tumour. Thus, DCIS found in MRI may be one of the reasons for overestimation of the size of the invasive tumour in some cases presented in this study [49].

Weatherall et al. found that the sampling error between pathological and MRI measurements decreased when the lesion diameter was obtained by averaging measurements made in three orthogonal dimensions [50]. This approach was not used in our study, but it may help to further improve the reproducibility and accuracy of our measurements.

CE was found to have a less dispersed data, and like MRI, it frequently underestimated size in comparison with pathology in our population. Clinical assessment was found to be less accurate for assessing complete responses, with a lower correlation with pathological measurements and 3 false-positive interpretations. Although, CE is essential, it is inherently subjective and cannot be validated as a definitive and exclusive assessment method.

This current study should be considered in the context of its strengths and limitations. The first limitation of our study was the small sample size. However, this cohort of patients represents a pilot sub-study of an ongoing trial. A second limitation was that the imaging-based measurements were finally reviewed in a consensus imaging interpretation, and thus, interobserver variability was not evaluated. The major strength is that there are no previous studies analysing MRI accuracy measuring treatment response in patients receiving NAAI therapy. Another strength is the consecutive prospective inclusion of patients. With an increasing use of NAAI therapy it is of particular interest to determine the most accurate of the current assessment methods to evaluate at the earliest the treatment effect.

In conclusion, our results showed that in postmenopausal patients diagnosed with LABC treated neoadjuvant with NAAI, the correlation between post-treatment MRI size and pathology was higher compared to the correlation between CE and pathology. MRI was found to be more accurate for estimating complete responses then clinical assessments. The results of this study provide further support for the benefit of a clinico-imaging preoperative assessment for evaluation of response, residual disease and the importance in deciding patient’s eligibility for BCS. Guidelines to correlate imaging and clinicopathological assessments, and further studies with larger sample sizes are needed.

## Electronic supplementary material

Below is the link to the electronic supplementary material.

Supplementary file1 (DOCX 13 kb)

## Abbreviations

3D: 3-Dimensional
AIs: Aromatase inhibitors
AJCC: American Joint Committee on Cancer
BCS: Breast-conserving surgery
BI-RADS®: Breast imaging-reporting and data system
CE: Clinical examination
CI: Confidence interval
DCIS: Ductal carcinoma in situ
DWI SSh-EPI: Diffusion-weighted imaging, single-shot, echo planar imaging
EPI: Echo planar imaging
ER: Oestrogen receptor
HER-2: Human epidermal growth factor receptor 2
LABC: Locally advanced breast cancer
LD: Longest diameter
MRI: Magnetic resonance imaging
NAAI: Neoadjuvant aromatase inhibitor
NET: Neoadjuvant endocrine treatment
O.d.: Once a day
PR: Progesterone receptor
r: Correlation coefficient
RECIST: Response evaluation criteria in solid tumours
TFE: Turbo field echo
TNM: *Tumour, node and metastasis*
TSE: Turbo spin echo
UICC: Union for International Cancer Control
