# Supplementary material for: Accuracy of breast MRI in patients receiving neoadjuvant endocrine therapy: comprehensive imaging analysis and correlation with clinical and pathological assessments
Source: Breast Cancer Res Treat. 2020 Aug 12;184(2):407–20. doi: 10.1007/s10549-020-05852-7 (PMC7599143; doi:10.1007/s10549-020-05852-7)
Supplement: Supplementary file 1 — Supplementary file1 (DOCX 13 kb) [file 10549_2020_5852_MOESM1_ESM.docx]

**Supplementary Table**

**Supplementary Table 1** The inclusion and exclusion criteria for this study

HER-2: human epidermal growth factor receptor 2, MRI: magnetic resonance imaging

| **Inclusion criteria** |
| --- |
| Patients with biopsy-proven locally advanced breast cancer |
| Large T2 tumours; primary tumours above 4 cm in diameter |
| Postmenopausal status |
| Estrogen receptor positive status |
| HER-2 negative status |
| No or distant limited distant metastasis |
| **Exclusion criteria** |
| Triple-negative breast cancer |
| HER-2 positive status |
| Life-threatening metastasis |
| Previous therapy for breast cancer within the last 12 months and/or medications that may interfere with endocrine therapy |
| Non-adherence to MRI examinations |
| Imaging quality does not meet the diagnostic requirements |
| Inconclusive CE |
| Inconclusive pathological examination |
| Refusal of surgery |
